# Supplementary material for: Are carbon emissions trading and green financial instruments synergistic? -Comprehensive quantitative research based on content analysis
Source: PLoS One. 2024 Mar 7;19(3):e0298601. doi: 10.1371/journal.pone.0298601 (PMC10919601; doi:10.1371/journal.pone.0298601)
Supplement: S1 Data — (ZIP) [file pone.0298601.s001.zip › Data/地级市Z_F工作报告.zip!/░▓╗╒/░▓╟∞╩╨/└┤╫╘╣1⁄2╓┌║┼í╛┬φ┐╦╩2╛▌═°í┐_2019.docx]

政府工作报告

——2019年1月8日在安庆市第十七届人民代表大会第二次会议上

市长  陈冰冰

各位代表：

现在，我代表市人民政府，向大会报告工作，请予审议，并请市政协委员和其他列席人员提出意见。

一、2018年工作回顾

刚刚过去的2018年，我们坚持以习近平新时代中国特色社会主义思想为指导，全面贯彻党的十九大和十九届二中、三中全会精神，在省委、省政府和市委的坚强领导下，坚持稳中求进工作总基调，认真落实新发展理念，深入实施五大发展行动计划，加快建设区域性中心城市，较好地完成了全年目标任务，经济社会加快转入高质量发展轨道。

过去的一年，我们克难攻坚，集中精力抓了以下重点工作：

**——加强经济运行调度。**面对错综复杂的宏观经济形势和经济下行压力加大的挑战，始终坚持逐月调度，在由市县乡三级干部参加的市委常委扩大会议上，有针对性地提出工业运行、项目建设、企业解困、营商安商等工作意见。从7月份开始，围绕“四督四保”“四送一服”，成立了12个督导组，开展了为期半年的经济工作督导专项行动，市政府坚持每半个月召开一次督导组长汇报会，市县两级1300多名干部共走访企业2140家、调度项目770个、解决问题1200多件。

**——推进省级以上开发园区改革。**借鉴筑梦新区体制机制创新的经验，进一步为园区赋权赋能。市经开区推行了管理、组织、分配、用人等全方位体制机制改革；市高新区在转隶原大观经开区凤凰片和托管山口乡的基础上，实施了山口片区整体开发和计划单列的园区体制机制设置；各县（市）区在推行一区多园之后，全面复制了市经开区的改革模式。

**——开展首位产业招商。**完善首位产业“十个一”方案设计，发起首位产业招商年活动，创新全员招商方式，改进北上广（深）招商推介的形式与内容，建立共引共建共享机制，加强过程管控和绩效考核。全市新签约亿元以上首位产业项目201个，实际到位资金179亿元，市经开区消费电子零配件产业园、桐城塑料机械制造、太湖光学离型膜、望江汽车类超细线路板等一批10亿元以上项目相继落户；新引进科研院所共建研发平台17个、人才团队26个。

**——聚力“三大攻坚战”。**一批突出环境问题得以解决，按时序要求推进中央和省环保督察反馈问题整改，整治“污乱散”企业1348家、畜禽养殖规模企业145家，拆除小水电站18座、沿江湿地水面围网养殖90多万亩，完成主城区十里大沟、谷桥大沟、大寨沟黑臭水体整治，实施天朋屠宰场关闭，推进集贤关区域生态覆绿，涉江豚自然保护区生态环境的18个入江排污口全部实现达标排放、长江码头泊位拆除18个、减船退渔258艘。精准脱贫有力有序，开展“四大攻势”行动，产业扶贫效果明显，土地增减挂钩工作全省领先，顺利完成8.52万贫困人口脱贫、101个贫困村出列的任务，岳西在全省国家级贫困县中率先通过高质量摘帽验收。金融生态环境稳步向好，深入推进国家社会信用体系示范市建设，扎实开展金融环境整治专项行动，涉金融债权和恶意逃废债案件办结4500多件，银行不良资产得到进一步化解。

**——加大棚户区改造力度**。2018年仅主城区已完成棚户区改造420万平方米、老旧小区改造180万平方米，惠及13.4万人；同时，全面抓紧保障性安居工程建设与分配，推动祥和南北苑、辉煌家园等一批遗留问题的妥善解决，多年未得到安置的群众约5390户1.6万人搬进了新居。

**——完成一批重大规划的编制。**面向安庆实际和未来发展，聘请国内顶级咨询机构，编制完成《安庆市林长制实施规划》《安庆市全域旅游发展规划》《国家养老服务改革试点地区规划》《职业教育集团化办学的规划意见》等，并强力推动付诸实施。

过去的一年，我们统筹推进经济社会发展，各项事业取得了全面进步：

（一）“六稳”目标基本实现。认真落实中央关于“稳就业、稳金融、稳外贸、稳外资、稳投资、稳预期”的要求，完善“4+X”产业政策体系，打出促发展的组合拳，保持了经济平稳较快发展。预计全市生产总值突破1900亿元，增长8%以上；财政收入308.9亿元，增长6.2%；社会消费品零售总额812亿元，增长14.8%；进出口总额104.8亿元，增长11.5%；全社会固定资产投资增长11.5%；新增贷款264亿元，增长15.5%；新增城镇就业8万人，农村劳动力转移就业6万人。

（二）产业新体系初具雏形。**大力发展战略性新兴产业和高新技术企业。**战略性新兴产业产值、高新技术产业增加值分别增长19%和20%，均居全省前列。**加快促进企业上市挂牌。**全市新增1家企业报会、1家企业报备，新三板挂牌企业达到22家、省股权交易中心挂牌企业达到307家。**持续推动首位产业发展。**首位产业产值达到1300亿元、增长20%，市经开区新能源汽车、市高新区化工新材料、桐城智能制造、望江和宿松纺织服装、迎江现代服务业等产业产值已过百亿，太湖功能性膜材料、怀宁新材料、潜山医药健康、岳西大健康和宜秀健康食品加工等产业正在形成集群集聚发展态势。

（三）美好大宜城建设步伐加快。完成城市建设投资57.5亿元，增长11%，建成区面积从95.2平方公里扩大到101.6平方公里。**交通基础设施加快完善。**新（改）扩建孝肃路、市府路等34条道路，独秀大道、迎宾大道、皖江大道全线贯通，五里庙和长风港一期码头改造顺利实施。**人居环境整治扎实推进。**人民路以南历史文化街区L型街改造的示范段即将运营，“四湖两河”截污工程完成，新（改）建排污管网100公里，新增城市绿化面积102万平方米，秦潭湖、石塘湖入湖口环境整治工程开工建设。**公共服务体系不断健全。**安庆会展中心、海吉星农产品物流园和皖西南快递产业园开工建设，安庆一中龙山校区建成，市一院龙山院区主体工程完工，成功举办第八届中国（安庆）黄梅戏艺术节，养老服务信息一体化平台投用，新增公共停车位1万个，新开通公交线路5条。

（四）生态环境保护更加凸显。**全力以赴抓好污染防治。**积极开展河湖清洁行动，地表水国控考核断面水质达到考核要求，县级以上集中式饮用水水源地水质达标率100%；积极开展固体废弃物集中整治，安庆静脉产业园一期开工建设，皖能中科二期建成运行；积极开展大气污染防治，主城区35蒸吨、县域10蒸吨以下工业燃煤锅炉全面取缔，主城区实现烟花爆竹禁限放，全市空气质量综合指数全省第四。**扎扎实实推进生态建设。**制定实施了《全面打造水清岸绿产业优美丽长江（安庆）经济带“1515”方案》，以“八大行动”构筑生态保护“三道防线”；推深做实了林长制，新增国家林业经济示范基地2个、省级现代林业示范区2个，完成人工造林和退化林修复20万亩，湿地保护面积157万亩、居全省第一；积极创建国家生态文明建设示范市，深入推进农村“三大革命”，组织开展自然保护区“绿盾2018”行动，岳西成为国家级生态文明示范县，潜山、迎江、宜秀成为省级生态文明示范县区。

（五）乡村振兴战略深入实施。**现代农业加快发展。**完成粮食生产功能区、重要农产品生产保护区划定工作，新增亿元以上产业化龙头企业20家，农产品加工业产值增长15%，新增省级以上示范合作社19家、示范家庭农场33家，培训新型职业农民5800人，新认证农产品“三品一标”213个，潜山舒席、怀宁蓝莓晋升国家地理标志产品，桐城、岳西成为全国电商百佳县。**农村环境持续改善。**下浒山水库下闸蓄水，华阳河蓄滞洪区安全项目前期工作顺利，治理中小河流10条，除险加固小水库27座，完成农村道路畅通工程2600公里，建成68个乡镇生活污水处理设施，完成5.2万户农村厕所改造，新建美丽乡村中心村110个，改造农村危房1.3万户，解决了5.8万农村人口饮水安全问题。**乡村治理更加规范。**圆满完成村（居）民委员会换届工作，建成126个民主法治示范村，岳西土桥村等4个村（社区）成为全省城乡社区协商试点示范单位。

（六）改革开放取得积极进展。**改革取得新成效。**深化职业教育改革，优化职教资源整合，安庆职业教育集团正式成立，产教融合、校企合作将迈出实质性步伐；深化“放管服”改革，全面推行“互联网+政务服务”，推进“一网、一门、一次”办理，全市政务服务融入全省“一张网”，网办事项达到95%；深化农村综合改革，完成300个村“三变”改革试点任务，建成覆盖全市的农村产权交易市场；深化医药卫生体制改革，大力推行现代医院管理制度，县域内首诊比例达到65%，分级诊疗的格局初步形成。**开放迈出新步伐。**合安九高铁建设全线推进，岳武高速东延线、德上高速桐城段开工建设，安庆至桐城等一级公路建成通车，全市铁路发送旅客549万人次，天柱山机场旅客吞吐量近50万人次，安庆B保中心进口总值位居全省4个同类平台第一，汽车整车进口口岸基本建成，安庆成为长三角城市经济协调会成员单位。

（七）民生工程不断提质提效。**努力提高民生保障标准。**城乡常住居民人均可支配收入分别增长8.5%和9.8%，全市基本养老保险参保率达到95%，被征地农民养老保障待遇标准提高60%，城乡低保、农村五保、特困人员保障全面提标，教师待遇得到全面提高和保障。**牢牢守住社会安全底线。**深入推进全国社会治安综合治理优秀市创建工作，扫黑除恶专项斗争扎实开展，“深重促”专项行动化解疑难复杂信访问题138件；加强重点领域安全监管，严密防控非洲猪瘟疫情，成功创建省食品药品安全城市，出台《安庆市危险化学品安全管理条例》，全市安全事故起数和死亡人数分别下降45.5%、21.2%。

（八）政府自身建设全面加强。坚持把学习习近平新时代中国特色社会主义思想作为首要政治任务，牢固树立“四个意识”，切实做到“两个维护”，坚决同以习近平同志为核心的党中央保持高度一致。扎实开展“讲严立”专题警示教育，推动“两学一做”学习教育常态化制度化。深入推进“七五”普法，重新修订《安庆市人民政府工作规则》，健全完善依法决策机制。自觉接受人大法律监督和政协民主监督，市人大代表议案建议、市政协委员提案办复率均为100%。严格落实中央八项规定，驰而不息纠正“四风”。立行立改省委巡视反馈问题，建立健全长效机制。注重各项工作统筹，妇女、儿童、老年人、残疾人权益得到保障，民族宗教、外事侨务、国防动员、国家安全、人防、地震、气象、涉台、保密、档案、地方志、红十字、关心下一代等工作取得新的进展。

各位代表！过去一年的成绩来之不易。这是省委、省政府和市委坚强领导的结果，是市人大、市政协和社会各界关心支持的结果，是全市人民齐心协力、共同奋斗的结果。在此，我代表市人民政府，向全市广大工人、农民、知识分子、干部，向驻宜解放军指战员、武警官兵、公安干警和消防救援队伍指战员，向各民主党派、各人民团体和社会各界人士，向关心支持安庆建设发展的中央和省驻宜单位、国内外友好人士、安庆籍在外地工作学习人员，向在我市创业发展的投资者、建设者，表示崇高的敬意和衷心的感谢！

在肯定成绩的同时，必须清醒地认识到，我们的工作还存在许多不足，发展还面临不少困难。主要表现在：工业化水平不高，外贸外资增长不快，科技创新能力不强，实体经济面临的问题仍较突出，园区转型升级任务艰巨，保持经济较快增长的压力较大；新型城镇化水平提速较慢，县域经济综合竞争力不强，城乡居民收入仍低于全省平均水平，民生领域还存在不少短板；政府工作人员思想观念、能力素质、担当精神还不能完全适应高质量发展要求，形式主义、官僚主义仍不同程度存在。对此，我们将采取有效措施，认真加以解决。

二、2019年工作安排

今年政府工作的总体要求是：以习近平新时代中国特色社会主义思想为指导，全面贯彻党的十九大和十九届二中、三中全会精神，按照省委、省政府的战略决策和市委“456”的总体部署，坚持稳中求进工作总基调，坚持新发展理念，坚持推动高质量发展，坚持以供给侧结构性改革为主线，坚持深化市场化改革、扩大高水平开放，在“巩固、增强、提升、畅通”上下功夫，加快推进工业化、城市化、区域化，持续打好三大攻坚战，进一步稳就业、稳金融、稳外贸、稳外资、稳投资、稳预期，保持经济社会健康发展。

今年经济社会发展的主要预期目标是：全市生产总值增长8%，财政收入稳步增长，社会消费品零售总额增长12%，全社会固定资产投资增长10.5%，城乡常住居民人均可支配收入分别增长8%和9%，居民消费价格涨幅控制在3%左右，城镇调查失业率控制在5%以内。

今年的重点工作是：

（一）聚焦工业化，着力提升首位产业的整体竞争力

按照水清岸绿产业优的要求，优化园区空间和产业布局，强化创新驱动，推动首位产业集群化、加速度发展。

**做大园区格局**。立足产业特色和功能定位，打造一流园区。**一是彰显比较优势**：市经开区充分发挥要素资源优势，利用好1.5万亩工业净地，加快投融资平台市场化改造，坚定不移推进首位产业集聚发展，做大龙头，延链补链；市高新区充分发挥山口片区的空间优势，遵循“1515”规范，高水平谋划专业分区，多规合一地建设化工新材料和医工医药的循环经济园区；市筑梦新区充分发挥“四新经济”的优势，着力打造基金小镇，力争再引进10家以上天使、创投及股权投资机构，构建服务全市、覆盖企业全生命周期的多元化股权投资体系，推动资本、技术、人才集聚，更好发挥孵化器、加速器作用；其他园区也必须尽快培育各自的产业发展长板。**二是坚持创新引领**：下大力气植入与高等院校、国家级科研院所共建的研发平台，推进产学研结合，实现科技成果就地转化，提高园区的核心竞争力。市经开区的新能源汽车研究院、高新技术企业孵化中心，市高新区的北化院化工新材料中试基地、中国技术交易所区域性化工新材料技术交易平台，市筑梦新区的增材制造、汽车轻量化等高新技术研发平台，迎江现代服务业研究院、大观环保产业研创平台、宜秀江南大学科技成果转化中心、桐城先进技术与产业创新研究院、怀宁新材料研究院、潜山生物医药研发平台、岳西中关村双创中心、太湖光学膜材料工程研究院、望江中国农科院长江科研中心、宿松纺织服装技术研究院，力争于年内实质性运营。**三是实现“腾笼换鸟”**：结合土地“四优四强”工作方案，开展土地清理专项行动，加快处置“僵尸企业”，依法合规地淘汰落后产能、落后工艺、落后企业，坚持以亩均投资强度和实际贡献论英雄，主城区释放4000亩、七县（市）释放3000亩园区闲置和低效用地，从而将要素资源配置给优质企业，使园区产业的首位度更高、带动性更强、生命力更旺。

**做实重大项目**。盯牢抓紧一批重大产业项目，加快形成现实生产力。**一督开工**：安庆石化的百亿技改项目、惠生（中国）投资公司和曙光集团的合作项目、日本爱信集团和环新集团的合资项目、普利制药、鼎泰智能制造、爱德马天然弱碱水、宁波热电联供等一批项目，必须启动实施。**二督投产**：江淮新能源汽车力争落实年产8万辆的计划，华茂全流程智能纺纱、华霆动力新能源汽车电池、力天增塑剂、晶凯集成电路电子、中建材高效单晶电池、岳西大数据中心、集友股份烟标生产线、金张科技功能膜工业园一期、旭众智能科技、森淼纤维素等项目，必须尽快明确投产时间表。**三督落地：**20万辆新能源整车、汽车热交换系统、子午线轮胎、驱动电机、环保仪器仪表、仲辰光电、申洲针织年产2000万件高档服装、宿松年产100万锭棉纺等一批在谈大项目，必须专班跟进、专人服务，争取早签约、早落地、早开工。

**做强专精特新**。培育一批细分市场的“小巨人”企业，引导中小企业走“专精特新”之路。**一是推进企业“升规”**：建立“小升规”企业培育目录，加强分类指导，加大政策支持，推动小微企业依托现有基础，加快做大做强，扩大市场份额，全市新增“小升规”企业100家以上。**二是推进企业“提质”**：鼓励企业运用互联网、物联网、云计算、大数据、人工智能等现代技术手段，创新生产方式，提高管理水平，支持企业开展产品、工艺和技术攻关，全市实施亿元以上智能化、绿色化技改项目100个以上，新增高新技术企业80家以上，争取更多的产业和企业进入省“三重一创”行列。**三是推进企业“入场”**：瞄准资本市场，加速企业股改，扩大直接融资，加快推进玉禾田上市，金张科技、华业香料、回音必等企业报会，新增5家以上企业新三板挂牌，力争100家以上企业在省股权托管交易中心科创板挂牌。

（二）聚焦城市化，着力提升中心城区的能级

围绕区域性中心城市定位，不断提升主城区的现代化程度和对周边地域的影响力、辐射力、服务力。

**加快城市更新。**围绕2020年主城区建成155平方公里的目标，推进“城市双修”，丰富城市内涵，提升城市品质。**一是启动重点片区开发建设：**启动以“安庆之星”为标志的滨江CBD建设，启动集贤关片区的“两区两园”改造，启动建设高铁新站区站房一期、站前广场、换乘中心等项目。**二是进一步完善城市交通网络：**加快推进中兴大道高架、安庆北互通迁移、长风港和皖河新港疏港铁路专用线、长风港二期、天柱山机场改扩建等项目建设，构建互联互通的综合交通体系；开工建设滨江大道、人民路东延伸线、勇进路大桥及山口段延伸线、中山大道西延伸段等项目，建成天柱山路西延伸线，完成菱湖南路、德宽路改造工程，打通文苑路、港口路等，构建内通外畅的城市骨干路网。**三是持续改善人居人文环境：**改造棚户区220万平方米、老旧小区156万平方米；开工建设人民路以南历史文化街区的沿江片区，建成投运人民路南侧的L型街区，实施大王庙、大观亭传统风貌街区修复；完成主城区雨污分流改造、石塘湖备用水源地生态环境整治；建成秦潭湖、神灵潭公园，实施潜江路、独秀大道等绿化景观提升工程，新增城市绿化面积120万平方米。

**改善商务条件。**注重补齐功能短板，增强城市承载能力。**一是加快完善现代物流体系：**依托市经开区B保中心平台、大力发展保税物流，依托大观区皖西南快递物流产业园、大力发展快递配送物流，依托迎江区运通达与路歌物流联盟等、大力发展工业企业第三方物流，依托宜秀区海吉星农产品物流园与百国庄园跨境电商城、大力发展冷链物流，并在此基础上，积极引进普洛斯（中国）等国际化的现代服务业集成运营商。**二是加快完善商业设施体系：**推进星级酒店建设，促使置地、绿地、恒大等在建的高标准酒店加快进度，提升城市大型商务活动保障能力和服务水平；推进会展中心建设，力争主体工程基本完工，服务会展经济；推进公共停车场PPP项目建设，新增公共停车位1万个；争取综合保税区获批并开工。**三是加快完善中介服务体系：**积极引进和培育为经济高质量发展提供专业化服务的金融证券、财务会计、工业设计、评估认证、信息咨询、检测检验等社会中介机构，鼓励设立区域性总部。

**优化公共服务。**大力推进文教卫体养游等领域开放合作，实现公共服务优质均等，多渠道扩大服务供给，满足消费需求。**一是提供优质的教育服务：**想方设法引进从幼儿到高中教育一贯制的国际学校；安庆一中龙山校区秋季正式招生，扩大一倍安庆一中的招生规模；职业教育集团实体化运作，完成院系调整、优化专业设置、整合教训资源，推进产教融合、校企合作，职业院校招生规模增长50%，加速加倍培养高素质技工人才；支持安庆师大、安庆医专、桐城师专、黄梅戏学院加强学科建设与人才引进。**二是提供周到的医卫服务：**市一院龙山院区投入使用，市中医院北城院区正式运营，谋划推进同济大学附属安庆医院尽早落地。**三是提供丰富的文体服务：**完善城区文体小广场布点和设施配套，加快“前言后记”书店的新项目建设，确保市博物馆5月18日对外开放，办好安徽省第三届健身休闲大会，谋划黄梅戏梦工厂项目。**四是提供多元的养老服务：**深化全国养老服务改革试点工作，居家、社区、机构+信息化平台的养老服务进一步完善，完成40个社区居家养老服务站提升改造，推进公办养老机构社会化运营，创新护养结合、医养结合模式，发挥市场在养老服务资源配置中的作用。**五是提供高质的旅游服务：**构建智慧旅游管理平台，落实全域旅游发展规划，挖掘主城区及周边旅游资源，打造行、游、住、吃、购、娱等融合发展的服务体系。

（三）聚焦区域化，着力提升县域经济统筹发展的水平

以增强县域经济实力为核心，促进区域协调发展，努力实现更高质量、更有效率、更加公平、更可持续的发展。

**全力攻坚转型发展**。按照供给侧结构性改革要求，更新发展观念，改进发展方式，推动产业迈向中高端。**一是以深化省级开发区改革为抓手，不断增强园区竞争力：**深耕园区“十个一”工程，强化要素协同，建成10个以上百亿级首位产业集群；坚持精准招商，围绕目标企业、产业链条、创新资源，主攻牵动性强、聚合度高的首位产业项目，加快引进和培育“独角兽”企业，每个园区新引进亿元以上项目不少于15个；坚持开放发展，提高经济外向度，全市进出口总额增长10%以上，外商直接投资增长20%以上；全力争先进位，所有园区在全省考核中进2位以上，在现有统计口径不变的情况下，桐城开发区的目标产值达到500亿元，怀宁开发区达到300亿元，宿松开发区达到250亿元，潜山、望江开发区达到200亿元，太湖开发区达到150亿元，岳西开发区达到120亿元，宜秀开发区达到100亿元，迎江开发区达到50亿元，大观开发区在完成新区规划建设的同时，实现稳步增长。**二是以推进乡村振兴战略为抓手，努力做好产业兴旺大文章：**深化农村综合改革，规范有序推进土地增减挂钩工作，完成300个行政村“三变”改革任务，力争土地使用权流转率达到55%以上，发展农业规模经营，壮大村级集体经济；调整优化产业结构，大力发展特色种养，提高有机农产品比重，提升品牌影响力和市场占有率；促进一二三产业融合发展，大力发展精深加工，积极培育龙头企业和新型农业经营主体，注重健全社会化服务体系，因势利导休闲农业、农村电商等新业态，新增亿元以上产业化龙头企业15家、省级示范合作社10家、省级示范家庭农场20家以上，培育新型职业农民5000人以上，农产品电商交易额达到25亿元以上；支持农民工返乡创业，新建创业园14个，带动群众就近就业4000人以上；因地制宜发展特色小镇，生产生活生态“三生融合”，宜居宜业宜游“三宜一体”，实现每个县（市）区培育2个以上特色小镇的目标；加快改善农村基础设施，建设3000公里“四好农村路”，落实引江济淮工程任务，推进华阳河蓄滞洪区安全项目，治理中小河流12条、除险加固小水库26座。**三是以帮助小微企业纾困为抓手，推动民营经济健康发展：**把功夫下在落实和用好国家、省支持民营经济发展的政策上，切实改善企业的税收、成本、营商环境，进一步畅通金融血脉，优化金融环境，防范金融风险，帮助民营企业解决融资难、融资贵问题，充分听取企业对政策执行的意见和建议，在市县配套政策上出实招，使其具有针对性和精准性；把企业家素质提升工程摆上突出位置，全年培训企业家1500人次以上，引导企业经营管理者增强信心，聚精会神办企业，遵纪守法搞经营，建立现代企业制度，压缩生产经营成本，加快科技创新步伐，提升核心竞争能力，进一步聚焦实业、做精主业。

**全力攻坚精准脱贫。**坚持把脱贫攻坚作为重大政治任务，深入推进“十大工程”，完成3.4万贫困人口脱贫、21个贫困村出列、望江和太湖2个贫困县摘帽，质量水平保持全省前列。**一是强化产业扶贫路径：**坚持“四带一自”的产业扶贫模式，完善利益联结机制，引导工商企业和各类市场主体与贫困村、贫困户建立股份合作、劳务合作与产销合作关系，持续增加贫困群众的经营性、财产性、工资性收入。**二是加强涉农资金整合：**采取项目化管理方式，确保资金精准高效使用，集中财力补短板，有针对性地化解一批贫困村的基础设施、环境整治、公共服务、产业发展等突出矛盾，切实改善贫困村和贫困户的生产生活条件。**三是巩固提升脱贫成效：**坚持扶贫与扶志扶技相结合，加大技能培训力度，致力于人有一技之长，强化扶贫对象动态管理，统筹落实贫困村与非贫困村、贫困户与边缘户的帮扶措施，进一步完善防范返贫的长效机制。

**全力攻坚污染防治。**以共抓长江大保护为统领，着力实施生态文明示范市建设重大工程。**一是实施濒危物种保护工程：**以国内一流水平的江豚自然保护区为目标，深入推进江豚自然保护区生态环境问题整治，聘请国内顶级专家科学论证江豚保护区规划方案，积极申报省级江豚自然保护区，建立保护管理的长效机制；切实加强国家和省级自然保护区管理，确保野生动植物种群数量稳定、濒危物种得到有效保护，推动自然保护区生态环境稳定向好。**二是实施河畅水清工程：**深入推进河长制、湖长制，强化河湖岸线空间保护利用，加强污水综合整治，认真实施农村黑臭水体治理，开展农村畜禽规模化养殖的试点工作，全市规模以上入河排污口全部实现达标排放，12个地表水国控考核断面水质优良率达到90%以上。**三是实施净土保卫工程：**推进固体废物减量化、资源化，完成静脉产业园一期工程和皖能中科一期项目的重建，扎实抓好垃圾焚烧发电布点项目的建设；大力推行绿色种养、健康养殖，实现化肥、农药使用量零增长，畜禽养殖废弃物综合利用率达到75%以上。**四是实施蓝天保卫工程：**取缔县域35蒸吨以下工业燃煤锅炉，全面实现工业污染源达标排放；加强扬尘污染综合防治，推进扬尘污染防治立法；加大汽车尾气、秸秆焚烧、餐饮油烟治理力度，加快淘汰高排放老旧柴油货车，全市秸秆综合利用率保持在90%以上；PM_2.5_、PM_10_年均浓度降幅和空气质量优良天数均高于全省平均水平。**五是实施岸绿景美工程：**深入推进林长制，实施沿江堤岸生态复绿补绿增绿工程，完成公路干线绿色长廊建设300公里、芭茅山整治6万亩以上，全市人工造林10万亩、退化林修复12万亩以上。**六是实施绿色产业发展工程：**大力发展全域旅游，打造“戏曲、研学、康养、乡村、山水”五大主题板块，提升安庆旅游美誉度和吸引力，旅游业收入增长15%以上；大力发展清洁能源产业，力争桐城抽水蓄能电站开工建设，岳西抽水蓄能电站完成前期工作。

**全力攻坚民生保障。**完善制度、强化措施，精心做好各项民生工作。**一是促进就业更加充分：**坚持就业优先，加强就业培训，全年新增技能人才1.2万人，开展职业技能培训2.1万人，新增就业4.4万人，农村劳动力转移就业5.6万人。**二是推动教育优质均衡：**争创国家中小学教育质量综合评价改革示范区；推进学前教育三期行动计划，适龄幼儿毛入园率达到90%；切实保障义务教育和职业教育，高中和中等职业教育普及率累计不低于98%。**三是提升医卫服务水平：**深化公立医院综合改革，加强基层医疗机构标准化建设，每个县（市）区至少创建1个乡镇二级医院，贫困村卫生室100%达标；推广家庭医生签约服务，签约率达到65%以上。**四是改善群众居住条件：**七县（市）完成棚户区改造340万平方米、农村危房改造3300户，全市新增商品房450万平方米以上，公租房竣工率、分配率均达到90%以上，建立健全住房市场体系和住房保障体系，压实责任“稳楼市”。**五是健全社会保障体系：**推进全民参保计划，全市基本养老参保率稳步提高，基本医疗保险实现全覆盖；推进城乡低保标准统筹，提高农村五保、困境儿童等特困人员保障服务水平；残疾人精准康复服务率达到75%；推进全国殡葬综合改革试点工作，公益性公墓覆盖率达到100%。**六是提高社会治理能力：**大力加强精神文明建设，扎实抓好公民道德建设工程，不断提高城乡居民素质；积极创建全国社会治安综合治理优秀市，深入实施“雪亮工程”，持续开展扫黑除恶专项斗争，健全完善立体化信息化社会治安防控体系，加大社区治理创新力度，完善网格化管理机制，提高基层治安能力；畅通信访渠道，规范信访秩序，切实维护信访形势稳定；加强食品药品安全监管，保障老百姓饮食用药安全；推进安全生产“铸安行动”常态化，持续开展安全隐患排查治理，切实防止重特大事故发生，安全事故起数和死亡人数“双下降”。**七是充分聚合各方力量：**统筹工青妇、民族宗教、外事侨务、涉台、国防动员、国家安全、人防、地震、气象、保密、科普、档案、地方志、红十字、关心下一代等各方资源，凝心聚力推动各项事业同步高质量发展。

三、加强政府自身建设

改革开放再出发，对政府自身建设提出了新的更高要求。我们必须始终保持积极向上、奋发有为的精神状态，把解放思想大讨论激发出来的蓬勃朝气，转化为争先进位的昂扬锐气，以时不我待、只争朝夕的工作姿态，以功成不必在我、建功必定有我的责任担当，努力建设人民群众满意的政府。

提高政治站位。做到思想上更自觉，更加深入地学习习近平新时代中国特色社会主义思想，在学懂弄通做实上持续用力，用以武装头脑、推动工作。做到政治上更自觉，更加牢固地树立“四个意识”，切实增强“四个自信”，坚决维护习近平总书记在党中央的核心、全党的核心地位，坚决维护党中央权威和集中统一领导。做到行动上更自觉，更加坚定地服从党的领导，坚持从政治上看问题、在大局中找方位，切实提升驾驭全局、统筹发展、狠抓落实的能力，确保党中央、国务院和省委、省政府及市委的决策部署不折不扣落实。

增强法治意识。做尊法学法守法用法模范，深入实施“七五”普法，完善宪法和法律学习制度，健全行政部门主要负责人出庭应诉制度，增强政府工作人员法治素养和法治能力。完善依法行政机制，强调有法必依、有章必循，规范办事行为，做到程序到位，坚持在法律法规的框架下开展工作，提高政府决策与施政的法治化水平。深化行政执法体制改革，推行相对集中行政处罚权和综合执法，审慎行使行政裁量权，严格规范公正文明执法。配合市人大常委会做好相关立法工作。自觉接受法律监督、司法监督和社会各方面监督。

端正政绩观念。强化群众意识，坚持执政为民，正确处理当前与长远、显绩与潜绩、速度与质量、经济发展与环境保护的关系，既做让老百姓看得见、摸得着、得实惠的好事，又做为长远发展作铺垫、打基础的实事。强化服务意识，高质量完成政府机构改革，进一步转变政府职能，深化“放管服”改革，全面推行一网通办。强化务实意识，不断加强政府组成部门领导班子能力建设，深入开展“建设中层、赢在执行”行动，切实改进公务员队伍工作作风，力戒形式主义、官僚主义，坚决反对重“痕”不重“绩”、留“迹”不留“心”、出“勤”不出“力”，坚持在加强学习中开拓思路眼界、在深入调研中破解发展难题、在跑部跑省中争取更大支持。强化廉洁意识，严格落实党风廉政建设责任制，认真履行“一岗双责”，深化廉政风险防控，加强执纪监督，健全规范权力运行机制，努力营造风清气正的政务环境。

各位代表！唯有实干方能梦想成真。让我们更加紧密地团结在以习近平同志为核心的党中央周围，在省委、省政府和市委的坚强领导下，锐意进取，扎实苦干，奋力谱写新时代五大发展美好安庆的新篇章，以优异成绩庆祝中华人民共和国成立70周年！
